# In vivo survival and differentiation of Friedreich ataxia iPSC‐derived sensory neurons transplanted in the adult dorsal root ganglia

**DOI:** 10.1002/sctm.20-0334

**Published:** 2021-03-18

**Authors:** Serena Viventi, Stefano Frausin, Sara E. Howden, Shiang Y. Lim, Rocio K. Finol‐Urdaneta, Jeffrey R. McArthur, Kwaku Dad Abu‐Bonsrah, Wayne Ng, Jason Ivanusic, Lachlan Thompson, Mirella Dottori

**Affiliations:** ^1^ Department of Biomedical Engineering The University of Melbourne Parkville Australia; ^2^ The Florey Institute of Neuroscience and Mental Health Parkville Australia; ^3^ The Murdoch Children's Research Institute Royal Children's Hospital Parkville Australia; ^4^ O'Brien Institute Department St Vincent's Institute of Medical Research Fitzroy Australia; ^5^ Department of Surgery The University of Melbourne, St Vincent Hospital Fitzroy Australia; ^6^ Illawarra Health and Medical Research Institute University of Wollongong Keiraville Australia; ^7^ Department of Paediatrics The University of Melbourne Parkville Australia; ^8^ School of Medicine Griffith University Gold Coast Australia; ^9^ Department of Neurosurgery Gold Coast University Hospital Southport Australia; ^10^ Department of Anatomy and Neuroscience The University of Melbourne Parkville Australia

**Keywords:** dorsal root ganglia, human pluripotent stem cells, sensory neurons, transplantation

## Abstract

Friedreich ataxia (FRDA) is an autosomal recessive disease characterized by degeneration of dorsal root ganglia (DRG) sensory neurons, which is due to low levels of the mitochondrial protein Frataxin. To explore cell replacement therapies as a possible approach to treat FRDA, we examined transplantation of sensory neural progenitors derived from human embryonic stem cells (hESC) and FRDA induced pluripotent stem cells (iPSC) into adult rodent DRG regions. Our data showed survival and differentiation of hESC and FRDA iPSC‐derived progenitors in the DRG 2 and 8 weeks post‐transplantation, respectively. Donor cells expressed neuronal markers, including sensory and glial markers, demonstrating differentiation to these lineages. These results are novel and a highly significant first step in showing the possibility of using stem cells as a cell replacement therapy to treat DRG neurodegeneration in FRDA as well as other peripheral neuropathies.

## Graphical abstract

This study is the first to describe transplantation of dorsal root ganglia (DRG) sensory neural progenitors derived from human pluripotent stem cells (hPSC) into adult rodent DRG tissue. Donor cells differentiated to neurons and glia in vivo and expressed DRG sensory markers. These findings are highly significant in supporting stem cell‐based therapies for treating peripheral DRG neuropathies, including Friedreich ataxia. Figure created using BioRender.com

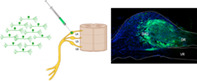

**Significance statement**

Within the nervous system, dorsal root ganglia (DRG) sensory neurons are responsible for sensing pain, touch, muscle movement, and tension. These neurons can degenerate in many different pathological conditions and diseases, including diabetes and Friedreich ataxia. In the present study, the authors describe transplantation of human DRG sensory neurons in adult rodents, within the DRG tissue region. The authors’ results are significant in supporting stem cell‐based therapies to treat peripheral DRG neuropathies, including Friedreich ataxia.

## 1 INTRODUCTION

The peripheral nervous system (PNS) is one of the primary and most significant sites of degeneration occurring in Friedreich ataxia (FRDA). FRDA is an autosomal recessive disease and it is considered the most common form of all inherited ataxias known to date.^1^ First symptoms are most commonly observed during childhood with an average age of onset of 10.0 ± 7.4 years.^2^ The various symptoms are strictly related to the severity of the disease and include absence of deep tendon reflex, dysarthria, areflexia of the lower limbs, diabetes mellitus, and cardiomyopathy. The neurological symptoms result from progressive degeneration of large sensory neurons (proprioceptive neurons) in the DRG and their axonal projection in the posterior columns, as well as from degeneration of the spinocerebellar and corticospinal tracts of the spinal cord.^3^ FRDA is due to the presence of a trinucleotide GAA repeat expansion in the first intron of the FRATAXIN (*FXN*) gene, causing reduced levels of full‐length FXN transcript and lower synthesis of the mitochondrial protein FXN. FXN is ubiquitously expressed and, within the mitochondria, helps the formation of iron‐sulfur cluster functioning as an iron‐chaperone.^4^ Low levels of FXN lead to a reduction in iron‐sulfur cluster synthesis with concomitant mitochondrial iron accumulation, mitochondria dysfunction, as well as an increased cellular oxidative stress.^5^, ^6^ All these events cause cell toxicity and cell death, particularly within the nervous system and cardiac tissue. Large sensory DRG neurons and cerebellar neurons are mainly affected within the nervous system. However, as clinical assessments are becoming more sensitive, several other neurological pathways appear to be affected, including the auditory and visual systems as well as speech.^7^, ^8^, ^9^ These findings suggest that neurodegeneration occurs within multiple neuronal cell types.

Although many breakthroughs have been made in unveiling some pathological mechanisms of the disease, others are still unclear. To date, most therapies are only aimed at slowing down the degenerative process and/or managing the symptoms. Several clinical trials are ongoing together with new studies underway to evaluate alternative future interventions.^10^, ^11^ In particular, gene therapy has attracted much interest thanks to its successful application in treating other disorders.^12^, ^13^, ^14^, ^15^ While drug and gene therapies are progressing to treat neurodegeneration and cardiomyopathy occurring in FRDA, cell replacement therapy remains an attractive treatment strategy to replenish some of the most severely affected mutant FXN cells.

Many transplantation studies have successfully demonstrated the ability of human donor cells to survive and functionally integrate in the central nervous system (CNS) leading to damaged neural tissue repair.^16^ Many of these studies utilized human fetal tissue, however, ethical concerns prompted exploration of alternative sources, particularly human pluripotent stem cells (hPSCs). Indeed, both mouse and human PSCs differentiated to neurons and transplanted in Parkinsonian rats showed comparable therapeutic potential.^17^, ^18^, ^19^ Transplantation of hPSC‐cell derivatives has also been examined for other neurodegenerative conditions and neurological disorders, such as animal models of epilepsy,^20^, ^21^, ^22^, ^23^ and spinal cord injury,^24^, ^25^, ^26^, ^27^ most of which showed promising results.

While many studies describe cell replacement in diseases affecting the CNS, less is reported for the PNS. Most research focuses on peripheral nerve regeneration and very few show cell replacement, particularly in the DRG. In 1997, Kozlova et al explored the in vivo properties of human DRG from embryonic donors in the cavity of DRG after its removal. The study showed the capacity of human embryonic sensory neurons to extend axonal fibers into the spinal cord and make connections in the gray matter area using blood vessels as cellular bridges.^28^ In 2006, Brännvall et al described transplantation of neural stem/progenitor cells derived from the cerebral cortex of embryonic mice in the adult rat DRG cavity, after the ganglion removal. Aggregates of donor cells placed in the DRG cavity showed expression for β3‐tubulin at 1 month post‐injection; however, no neuronal cells were observed in the long‐term study. Similar results were observed after transplantation of dissociated neural stem cells in the intact DRG.^29^ Other studies showed transplantation of mesenchymal stem cells into the DRG for investigating their therapeutic potential to treat pain through release of cytokines and chemokines.^30^, ^31^ In 2015, Hoeber et al analyzed the recovery of sensorimotor functions after dorsal root avulsion in mice.^32^ They placed hESC‐derived neural progenitor spheres along the spinal cord with access to the avulsed areas of L3‐L5 spinal cord. Immunostaining revealed that transplanted human neural progenitors differentiated to neurons and glia. However, donor‐derived neurons did not show connections with the intrinsic spinal cord circuitries because they were unable to reach that region. Nevertheless, improvement in the animal's sensorimotor functionality was observed in behavioral functional tests, suggesting partial regeneration of sensory innervation into the spinal cord. Similar studies have been performed using rat boundary cap neural crest stem cells (bNCSCs) whereby they were placed in the dorsal root‐spinal cord junction following dorsal root avulsion injury.^33^ The presence of transplanted cells in the proximal part of the dorsal root was observed along the dorsal regions of the spinal cord. In contrast, no donor cells were found in the spinal cord of transplanted animals with intact dorsal roots, suggesting that the migration of bNCSCs occurs in response to injured tissue.

Earlier work from our laboratory has provided the only evidence of cell replacement therapy to treat FRDA available to date.^34^ We showed that FRDA iPSC‐derived neural progenitors transplanted into the cerebellum of adult rats survived, integrated, and differentiated within the host tissue. Grafted cells expressed neuronal markers NeuN and Tbr1, the glial marker GFAP, as well as markers of immature and mature oligodendrocytes, Olig2 and APC, respectively. These results are consistent with long‐term survival and integration of FRDA iPSC‐neuronal derivatives in the adult nervous system.

Genetic correction of diseased iPSCs enables cell replacement therapies as an alternative therapeutic approach for treating FRDA disease. Here we examine the capacity of transplanted hESC and FRDA iPSC‐derived sensory neural progenitors to mature and integrate within the adult DRG in vivo. Our findings show in vivo neuronal and glial differentiation of donor hESCs and FRDA iPSCs, whereby grafted cells express markers of nociceptor, mechanoreceptor, and proprioceptor sensory neuronal subtypes. These studies are the first to report transplantation of hPSC‐derived sensory neurons into the adult DRG and the outcomes of these analyses are valuable for further investigating novel therapies aimed at ameliorating FRDA.

## 2 MATERIALS AND METHODS

### 2.1 Generation of a GFP‐FRDA iPSC line

HPSC lines were used in accordance with the guidelines and regulations of Melbourne School of Engineering and OHS and with the approval of the University of Melbourne Human Research Ethics Committee (Approval no. 1545384 and 1545394). Reprogramming of FRDA patient‐derived fibroblasts to generate “FA10” iPSC was approved by University of Melbourne Human Ethics Committee (0829937) and conducted using the published protocol.^35^ FRDA iPSC cell line, “FA10,” was maintained as bulk in feeder‐free conditions on vitronectin as previously published.^36^ Pluripotency of FA10 cells was checked by expression of pluripotency markers (see Supplementary Figure S1) and differentiation toward germinal layers (see Supplementary Figure S1).

The FRDA‐iPSC line (FA10) was electroporated with the T2K‐GFP vector in which GFP was expressed under the control of CAG promoter (see Supplementary Figure S2). GFP‐positive FA10 iPSCs were identified 3 days after electroporation (see Supplementary Figure S3A) and manually isolated under epifluorescence illumination and expanded (see Supplementary Figure S3B), until formation of a GFP‐expressing colony (see Supplementary Figure S3C). GFP‐FA10 cells retained uniform and robust GFP expression after in vitro differentiation toward the sensory neuronal lineage. In fact, high levels of GFP expression were found during NSPs formation, which were maintained until in vivo transplantation (see Supplementary Figure S3D).

### 2.2 Culture and differentiation of hESCs and FRDA iPSCs

Human ES cell line, H9, and human FRDA iPSC cell line, FA10, were maintained as bulk culture in feeder‐free conditions on vitronectin (StemCell Technologies) coated flasks (Corning) using TeSR‐E8 basal medium (StemCell Technologies). Both cell lines were differentiated following the protocol for sensory differentiation as previously described.^36^, ^37^, ^38^ For induction, both H9 hESC and FA10 iPSC cell lines were plated onto freshly laminin (Invitrogen)‐coated organ culture plates and cell culture media changed every 2 days. On the fifth day, cells were enzymatically detached using 0.5 mM EDTA (Life Technologies) and cultured in suspension. The generated NSPs were cultured for further 5 days, supplemented with basic fibroblast growth factor 2 (FGF2) (20 ng/mL, Peprotech) and BMP2 (10 ng/mL, R&D system) to form NSPs consisting of neural crest progenitors using the published protocol.^36^ Following 5 days, NSPs were gently dissociated and plated on poly‐d‐lysine (Sigma) and laminin substrates at a density of 3 NSPs per coverslip in 24 well cell culture for immunostaining. For RT‐qPCR experiments, 5‐day old NSPs (9 NSPs per well of a 24‐well cell culture plate) were plated per week, for a total of 3 weeks. They were cultured further for up to 3 weeks in neural basal medium supplemented with NGF (10 ng/mL, Peprotech), NT3 (10 ng/mL, Peprotech), BDNF (10 ng/mL, Peprotech) and Y27632 (25 μM, Tocris). Media change was performed every second day and cells were either fixed and prepared for immunostaining or harvested and processed for RT‐qPCR analyses every week.

For transplantation, 3 week old NSPs were incubated for up to 10 minutes in accutase solution (Sigma‐Aldrich) and gently mechanically dissociated into a single cell suspension. Cell were then counted and resuspended at 6 × 10^4^ cell/μL in HBSS without Ca^2+^ or Mg^2+^, supplemented with 0.05% DNase. The cell preparation was stored on ice for the entire duration of the surgery.

### 2.3 Gene expression analyses

For RT‐qPCR analyses, cells were harvested and PureLink RNA Mini Kit (Life Technologies) used for total RNA extraction in a dedicated working area and carried out according to manufacturer's instruction. Quality of RNA was examined on NanoDrop 2000 Spectrophotometer (Thermo Fisher Scientific). RNA samples A_260/280_ ratio ranging from 1.98 to 2.05 were processed for cDNA synthesis. One microgram of RNA was used to synthesize first‐strand cDNA with random primers using SensiFAST cDNA Synthesis Kit (Bioline). The quantification of every mRNA was carried out by real‐time quantitative PCR and performed with TaqMan Universal Master Mix (Applied Biosystems). Relative levels of each transcript were normalized for the endogenous controls ELF1, GAPED and HMBS. Gene Expression is presented using the ‐2^ΔΔCt^ method. The specific probes (Life Technologies) that have been used are as follows: *BRN3A (POU4F1)* (Hs00366711_m1), *CALB1* (Hs010077197_m1), *ELF1* (Hs00152844_m1), *FAM19A1* (Hs00405421_m1), *GAPDH* (Hs02758991_g1), (*HMBS* (Hs00609297_m1), *ISLET1* (Hs00158126_m1), *LDHB* (Hs00929956_m1), *NECAB2* (Hs00332810_m1), *TRKA (NTRK1)* (Hs01021011_m1), *TRKB* (*NTRK2)* (Hs00178811_m1), *TRKC (NTRK3)* (Hs00176797_m1), *PLXNC1* (Hs00194968_m1), *PRPH* (Hs00986946_g1), *PVALB* (Hs00161045_m1), *RET* (Hs01120030_m1), *S100β* (Hs00902901_m1), *SPP1* (Hs00959010_m1), *STT* (Hs00356144_m1), *TAC1* (Hs00243225_m1), *TRPV1* (Hs00218912_m1), *TH* (Hs00165941_m1), *VGLUT3* (Hs00900423_m1).

### 2.4 Statistical analysis

Statistical analysis was performed using GraphPad Prism 8 software and data were presented as mean with error bars representing SE of mean (SEM). Statistical significance was evaluated using independent groups (unpaired) two‐tailed *t*‐tests for expression of each marker at 1 and 3 weeks of differentiation in both hESC and FRDA‐derived sensory neurons.

### 2.5 Animals

The use of animals in this study conformed to the Australian National Health and Medical Research Council's published Code of practice for the Care and Use of Animals for Science Purposes (2013), and experiments were approved by the Florey Neuroscience Institutes Animal Ethics Committee (Ethics no.: 15‐053‐FINMH).

Animals were housed in individually ventilated cages on a 12 hours light/dark cycle with ad libitum access to food and water. Rats aged ≥10 weeks, both Sprague‐Dawley and athymic strains, were included in the study. Females were used over males for housing purposes, given there is no evidence to suggest any sex‐effect on the outcome of the experiment.

### 2.6 Surgical procedure

The surgical procedures were done using a stereotaxic frame (Kopf, Germany) and a spinal adapter (World Precision Instruments). Prior to surgery, animals were anesthetized with isoflurane (5% at 1 L/min) and kept under anesthesia for the duration of the surgery (2% at 1 L/min). All animals were intraperitoneally administered an analgesic (meloxicam, 3 mg/kg). For transplantation into the DRG, a midline incision was performed through the skin and fascia, and then the muscles were dissected and retracted laterally to expose the lower lumbar vertebral column. A hemi‐laminectomy was performed to expose the spinal cord dura and the attached nerve root and right L4/L5 DRG. A volume of 0.5 μL containing 3 × 10^4^ or 5 × 10^4^ cells were injected into the DRG. Cells were delivered via a borosilicate glass capillary (Harvard instruments) connected to a 5 μL Hamilton micro‐syringe to ensure discrete delivery to the targeted DRG region and minimize physical damage. In addition, the administration rate was set at 0.5 μL/min and the glass capillary left in place for 5 minutes in order to prevent backflow. The cells were resuspended before each injection and kept on ice for the duration of the surgery. Grafted animals were maintained and monitored for 2 weeks (n = 6 for ENVY and n = 3 for mCherry, n = 6 for FA10‐GFP cell line, respectively) 6 weeks, and 8 weeks (n = 3 for FA10‐GFP cell line).

### 2.7 Pharmacological immunosuppression

Injectable immunosuppressant preparations were made fresh and kept at 4°C for 1 week. Cyclosporin A (Labseeker) was first dissolved in absolute ethanol and then emulsified in olive oil. Immunosuppressant was administered daily (10 mg/kg) starting from 5 days prior to surgery. Immunosuppressant was administered to animals by subcutaneous injection. Treatment with the compound continued for the duration of the study.

### 2.8 Tissue collection and preparation

Grafted animals were sacrificed by terminal dose of pentobarbitone (100 mg/kg; Virbac, Peakhurst, Australia) and transcardially perfused with paraformaldehyde solution (PFA, 4% in 0.4 M phosphate buffer). The injected DRG with the attached nerve roots, ventral root, spinal cord, and sciatic nerve were dissected and processed for sectioning. Dissected tissues were transferred to 20% sucrose PBS solution overnight, except for spinal cords that were additionally post‐fixed in PFA for a further 2 hours. DRGs were transferred to a Tissue‐Tek Cryomold Biopsy (block) filled with OCT compound and quickly frozen with an ethanol bath and dry ice. The other tissues were embedded into porcine gelatine (10% gelatine in 0.1 M PBS: Sigma‐Aldrich) in order to facilitate cuttings. For sectioning, OCT blocks were sectioned with cryostat into 35 μm thick sections, while gelatine blocks were cut into 30 μm thick sections on a freezing microtome (Leica, Wetzlar, Germany).

### 2.9 Immunostaining and imaging

For immunostaining, cells cultured on coverslips were fixed with 4% PFA on ice for 10 minutes. Cells and DRG tissues were permeabilized for 15 minutes at room temperature using 0.2% triton‐X100 solution. Incubation with primary and secondary antibodies was performed in 10% normal donkey serum (Millipore)/ phosphate buffer saline DPBS (Invitrogen) blocking solution, overnight at 4°C and for 1 hour at room temperature, respectively.

Free‐floating immunostaining for sciatic nerve and spinal cords were performed on a 1:9 series for fluorescent staining in order to determine viability, differentiation, and integration of donor hPSC‐progenitors into the host tissue. Sections were exposed to the primary antibodies in blocking solution (2% donkey serum, 0.3% Triton X‐100 and PBS) on a rocking platform at room temperature overnight.

The following primary antibodies were used: hCALB1 (goat, 1:500, R&D systems, AF3320), CGRP (mouse, 1:100, Abcam, ab811887), FAM19A1 (rabbit, 1:100, Atlas Antibodies, HPA013407), GFAP (rabbit, 1:600, Dako, Z0334), GFP (chicken, 1:1000, Millipore, AB16901), hMitochondria (mouse, 1:1000, Abcam, ab92824), NECAB2 (rabbit, 1:1000, Atlas Antibodies, HPA013998), NF200 (mouse, 1:2000, Sigma‐AldricH, N0142), hNuclei (mouse, 1:200, Millipore, MAB1281), hPLXNC1(mouse, 1:200, R&D systems, MAB3887), hPRPH (mouse, 1:200, Abnova, H00005630‐M02), PRPH (mouse, 1:500, Chemicon International, MAB1527), PV (sheep, 1:500, R&D systems, AF5058), RFP (rabbit, 1:500, Rockland, 600‐401‐379), hSPP1 (goat, 1:500, R&D systems, AF1433), SST (rat, 1:100, Millipore, MAB352), TRPV1 (rabbit, 1:1000, Novus Biologicals, NB120‐3487), hTRKA (goat, 1:200, R&D systems, AF175), hTRKB (mouse, 1:200, Novus Biologicals, NBP1‐47898), TRKB (mouse, 1:500, R&D systems, AF1494), hTRKC (rabbit, 1:250, Thermo Fisher Scientific, 701985), TRKC (goat, 1:500, R&D Systems, AF1494), TH (rabbit, 1:2000, Novus Biologicals, NB300‐109), TUBB3 (chicken, 1:2000, Abcam, ab41489), TUBB3 (mouse, 1:500, Merch, MAB1637). Species‐specific secondary antibodies were diluted in blocking solution and added to the cells for 1 hour at room temperature. Alexa Fluor 488 and 568 conjugated anti‐goat IgG, Alexa Fluor 568 and 647 conjugated anti‐mouse IgG, Alexa Fluor 488 and 568 conjugated anti‐rabbit IgG, Alexa Fluor 488 conjugated anti‐rat IgG and Alexa Fluor 488 conjugated anti‐sheep IgG were used as secondary antibodies at a final concentration of 1:1000 (Life Technologies), except for Alexa Fluor 488 conjugated anti‐chicken IgY (IgG) (1:200, Jakson ImmunoResearch, 703‐545‐155). Tissue sections on rocking platform were incubated with secondary antibodies at room temperature for 2 hours. Nuclei were visualized using 4′,6‐diamidino‐2‐phenylindole, dihydrochloride (DAPI) counterstain (1 μg/mL final concentration, Sigma‐Aldrich). Samples were mounted onto glass slides using moviol mountant or Dako Fluorescent Mounting Medium (Agilent Technologies). Each experiment included in parallel immunostaining of samples with negative controls. Image capture was performed using a Nikon A1R confocal microscope, a Zeiss 780 confocal microscope or Zeiss Observer z1 fluorescence microscope.

### 2.10 Quantification of cell numbers within the grafts

A 1:15 series of sections immuno‐labeled for mCherry or GFP were used to quantify DRG volumes in transplanted animals at 2 and 8 weeks post transplantation. A Leica (DM600) microscope equipped with a motorized X‐Y stage was used to capture photomontages of whole DRG sections. The whole DRG area of every consecutive section in a 1:15 series was measured. Cell counting was performed in three independent fields of view (20× objective) within the graft, as verified by fluorescent reporter expression. Graft volumes were calculated from the sum of the area, defined by fluorescent reporter signal, the section thickness and series interval according to the Cavalieri's principle (1996). Total number of TRKA^+^, TRKB^+^, and TRKC^+^ cells within the graft were counted based upon mCherry (2 weeks transplanted animals) and GFP (8 weeks transplanted animals) immunoreactivity from three fields of view captured at 20× magnification using a Carl Zeiss Axio Observer Z.1 epifluorescence microscope.

## 3 RESULTS

### 3.1 Phenotypic characterization of FRDA‐derived sensory neuronal subtypes

**FIGURE 1:**
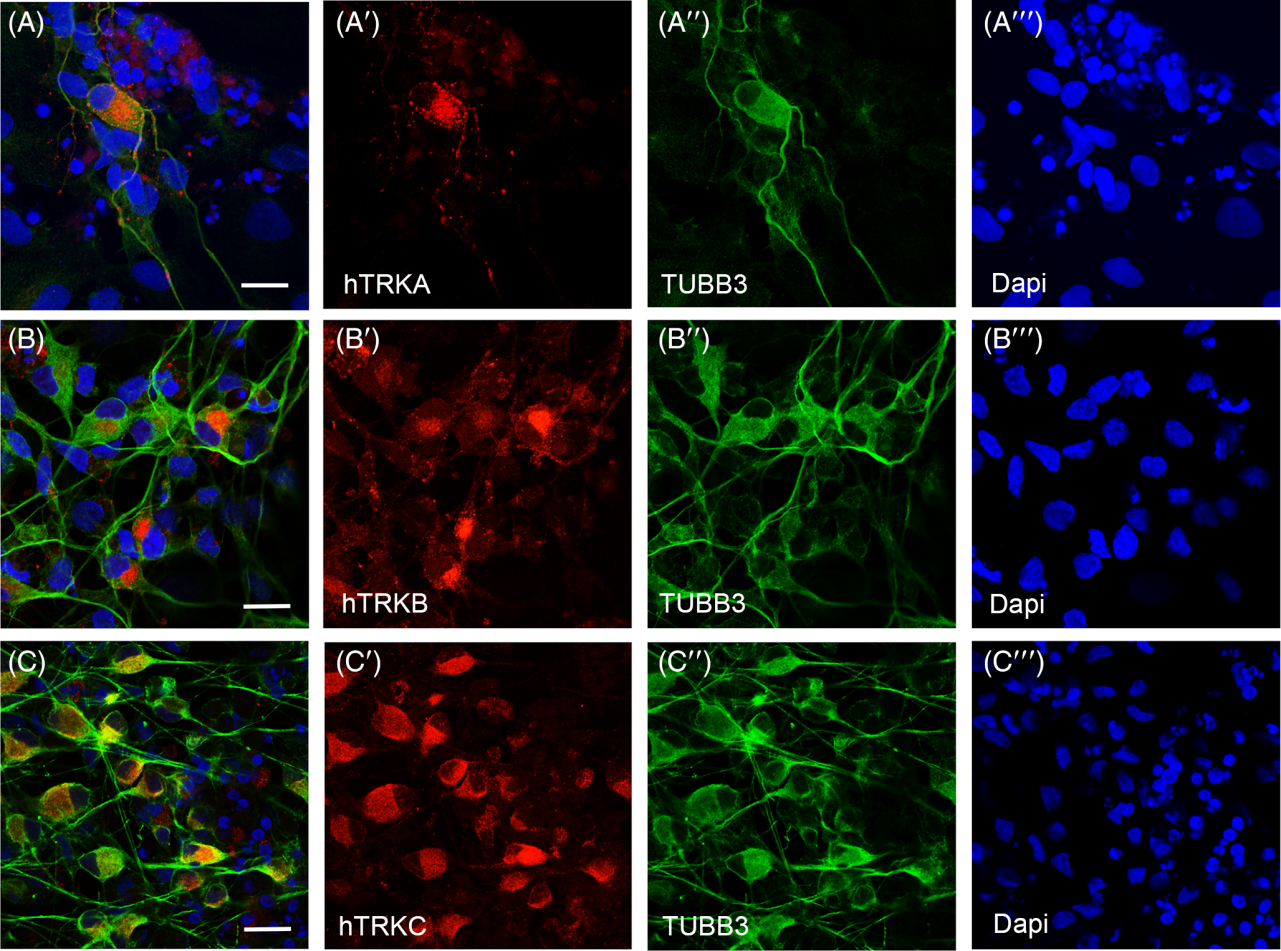
FRDA iPSC‐derived sensory neurons showing expression of markers for the three major subpopulations of DRG sensory neurons. FRDA‐derived cells differentiated to sensory neuronal progenitors show expression of neuronal marker TUBB3 (A, A″, B, B″, C, C″, green) and TRKs receptors (A, A′, B, B′, C, C′, red). Neurons positive for nociceptive marker TRKA (A, A′, red), for the mechanoreceptive marker TRKB (B, B′, red), proprioceptive marker TRKC (C, C′, red) are shown. Nuclei are shown in blue (DAPI). Scale bars = 20 μm. TUBB3, β3‐tubulin

Our previous studies described generation of functional DRG‐like sensory neurons from hESCs, and showed the cultures expressed markers associated with different subtypes of DRG mechanoreceptors, proprioceptors and nociceptors.^37^ Similar to our analyses of hESC‐derived sensory neurons, we differentiated FRDA iPSCs to sensory neurons and investigated the expression of DRG sensory neuronal subtype markers as described by Usoskin and colleagues.^39^ Immunostaining analyses performed on differentiated FRDA iPSCs show cells positive both for β3‐tubulin and TRKA, TRKB or TRKC receptors (Figure 1), suggesting the presence of three major DRG neuronal subtypes, nociceptors, mechanoreceptors and proprioceptors, respectively.

**FIGURE 2:**
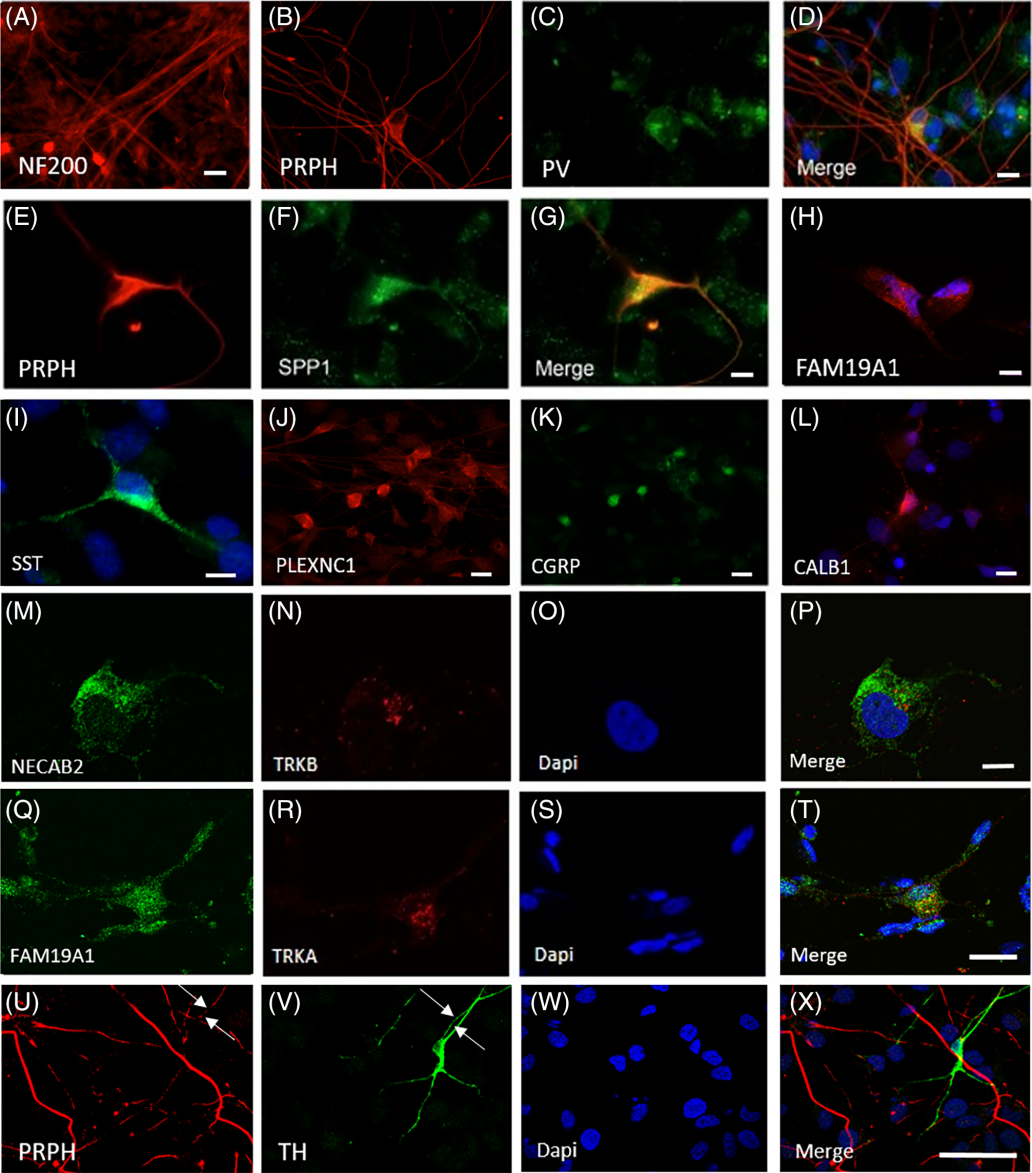
Expression of markers of nociceptors, proprioceptors, and mechanoreceptors subtypes in FRDA iPSC‐derived sensory neuronal cultures. Differentiated cultures show cluster of neurons expressing NF200 (A). Neurons co‐expressing both PRPH (B, D) and PV (C, D) and PRPH (E, G) and SPP1 (F, G) were found. Expression of FAM19A1 (H) and STT (I) was observed. Group of neurons co‐expressing PLXNC1 (J, red) and CGRP (K, green). Cell positive for CALB1 (L) was detected. Co‐expression of NECAB2 (M, P, green) and TRKB (N, P) was also observed in differentiated cultures. Cells positive for FAM19A1 (Q, T) and TRKA (R, T). C‐LTMRs neuron positive both for PRPH (U, X, red, arrowed) and TH (V, X, green, arrowed). Nuclei are shown in blue (DAPI). Scale bars = (A, T, J, K) 20 μm, (D, G, H, I, L, P) 10 μm, (X) 50 μm

Clusters of FRDA cells positive for neurofilament 200 (NF200), a marker for large DRG sensory neurons such as mechanoreceptors and proprioceptors, were also identified (Figure 2A). In particular, we observed presence of cells positive both for parvalbumin (PV) and peripherin (Figure 2B‐D) and cells co‐expressing osteopontin (SSP1) and peripherin (Figure 2E‐G), which are characteristic of proprioceptive neurons. Expression of a family with sequence similarity 19 A1 (FAM19A1), a marker that is expressed both by myelinated mechanoreceptive and myelinated peptidergic nociceptive neurons, was also observed (Figure 2H). Calbindin1 (CALB1) positive cells were also observed (Figure 2L). Cells showing co‐expression for the markers N‐terminal EF‐hand calcium binding protein 2 (NECAB2) and TRKB, which have been shown to identify different mouse DRG mechanoreceptive subtypes (Figure 2M‐P) were also observed. Expression of somatostatin (SST), which is typically expressed by nociceptors, may suggest the presence of unmyelinated nonpeptidergic nociceptors within the cultures (Figure 2I). We also detected cells positive both for plexin C1 (PLXNC1) and calcitonin gene related‐peptide (CGRP) (Figure 2J,K), markers that are expressed both by a specific subtype of nonpeptidergic neurons and unmyelinated peptidergic neurons, respectively. Positivity for FAM19A1 was detected in combination with TRKA marker (Figure 2Q‐T), suggesting differentiation of FRDA cells to peptidergic neurons. Lastly, a few cells tyrosine hydroxylase (TH) positive cells were identified to co‐expressed peripherin (PRPH), potentially indicating presence of unmyelinated C‐fiber low threshold mechanoreceptor neurons (Figure 2U‐X).

Expression of DRG sensory markers in neurons derived from hESC and FRDA iPSC cells was further supported by RT‐qPCR analyses (see Supplementary Figure S4). Transcripts levels of all markers analyzed remained constant from week 1 to 3 of hESC sensory neuronal differentiation. Instead, results from RT‐qPCR analyses performed on FRDA‐derived sensory neurons not only confirmed expression of markers shown by immunostainings, but also revealed expression of additional sensory markers at the RNA level. In addition, we observed a statistically significant increase in expression of some markers at 3 weeks of differentiation compared with week 1 differentiation. In particular, higher expression at 3 weeks of differentiation was identified for *CALB1*, *PLXNC1*, *TAC1*, *VGLUT3*, and *RET*. Of these markers, *CALB1* is expressed in mechanoreceptive neurons and *RET* both in mechanoreceptors and nociceptors. Instead, *PLXNC1*, *TAC1*, and *VGLUT3* are markers typically expressed by nociceptive neurons. Interestingly, decreased expression at 3 weeks of differentiation was shown for both markers expressed by proprioceptive neurons, *PV* and *SPP1*, which are the degenerating cells in FRDA patients. However, their decreased expression was not statistically significant. Nevertheless, more analyses are required using mutiple control and FRDA iPSC lines in order to determine whether FRDA iPSC lines show a more restricted capacity in sensory neuronal differentiation.

Overall, the expression profile analyses revealed the presence of cells that show positivity for a variety of DRG sensory neuronal markers of nociceptor, mechanoreceptor, and proprioceptor subtypes. These results suggest that FRDA iPSC are capable of differentiating to most, if not all, DRG sensory neuronal subpopulations in vitro.

### 3.2 Survival, localization, and differentiation of human ESCs‐derived sensory neurons 2 weeks after transplantation

**FIGURE 3:**
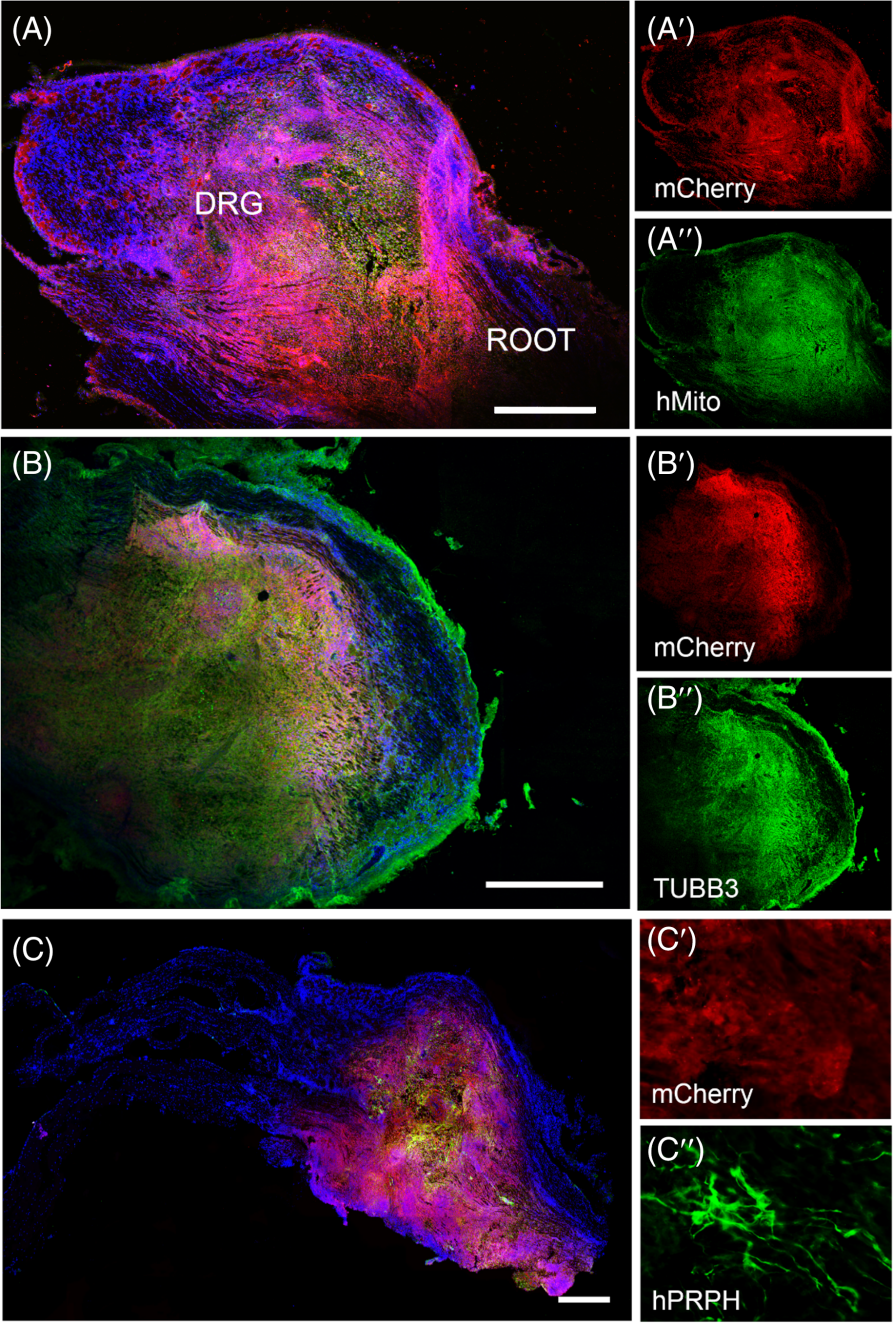
Presence of hESC‐derived sensory neurons in the DRG after 2 weeks post‐transplantation. (A) Image shows hESC‐derived donor cells (mCherry) in the DRG. mCherry‐positive cells (A′, red) and human‐specific mitochondria marker (A″, green) are co‐expressed. (B) HESC‐derived cells show expression of neuronal markers, TUBB3 (B, B″, green) and hPRPH (C, C″, green). Magnification of (C) showing (C′) mCherry and (C″) ^+^ PRPH^+^ cells, respectively. Nuclei are shown in blue (DAPI). Scale bars: (A, B, and C) 500 μm. hPRPH, human peripherin; TUBB3, β3‐tubulin

**FIGURE 4:**
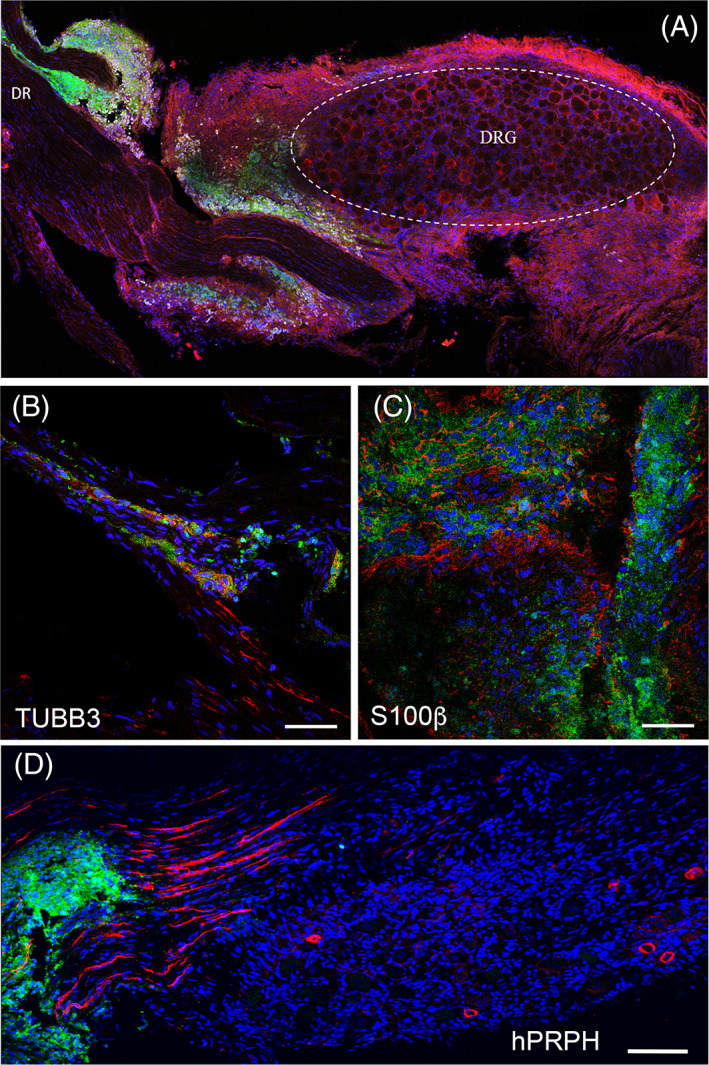
Transplantation and characterization of hESC‐GFP (ENVY)‐derived donor cells both within and around the DRG rat tissue 2 weeks post‐transplantation. (A) ENVY‐positive cells (green), osteopontin (SPP1, red) and human nuclei (hNuclei, white). (B) HESC‐derived cells show expression of neuronal marker TUBB3 (B, red) and glial marker S100β (C, red) as well as positivity for sensory markers hPRPH (D, red). Nuclei are shown in blue (DAPI). Scale bars: (A) 100 μm, (B, C) 50 μm, (D) 100 μm. DR, dorsal root; hPRPH, human peripherin; TUBB3, β3‐tubulin

We next examined whether hPSC‐derived sensory progenitors were capable of surviving and maturing in vivo in the adult DRG regions. Rats of ≥10 weeks of age were transplanted in the DRG region at the level of L4 with 3‐week‐old sensory neural progenitors differentiated from reporter GFP‐ or mCherry‐ hESC lines. The animals were sacrificed at 2 weeks post‐transplantation and histological analyses of DRG tissues were performed. Expression of the reporter hESC lines, GFP and mCherry, were uniformly maintained throughout the grafted DRG tissue, allowing us to perform analyses of the graft composition (Figure 3). Co‐immunostainings using human specific antibodies, such as human mitochondria or human nuclei, were also conducted to confirm presence of human transplanted cells (Figures 3A and 4A). Cell counts of the graft containing mCherry donor cells found a 3.35‐fold increase in mCherry positive cells relative to the cell number injected (5 × 10^4^ cells).

**FIGURE 5:**
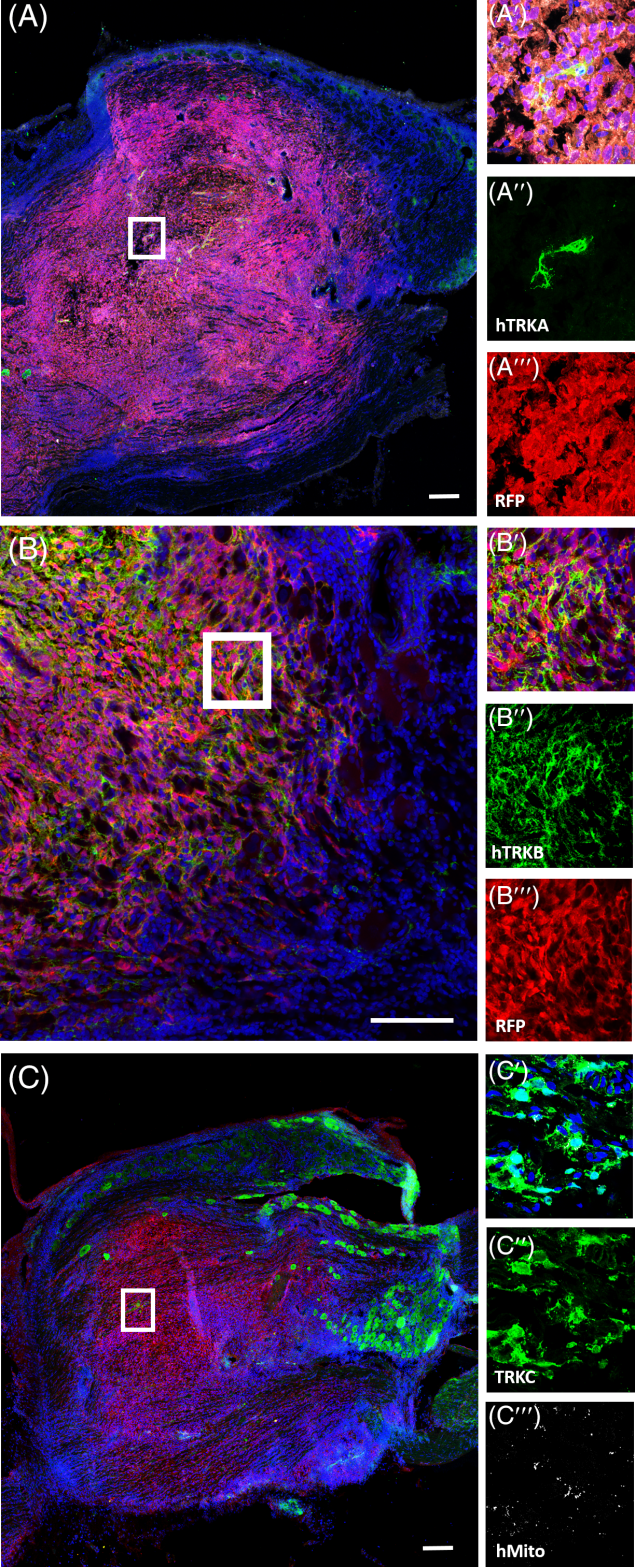
Expression of TRK receptors in hESC‐derived cells 2 weeks post‐transplantation in adult rat DRG. mCherry cells (red) positive for hTRKA (A, A′, A″, A″′, green), hTRKB (B, B′, B″, B″′, green), and TRKC (C, C′, C″, C″′, green). Transplanted cells were also positive for human mitochondria marker (C, C′, C″′). Nuclei are shown in blue (DAPI). Scale bars: (A, C) 200 μm, (B) 100 μm

Immunostaining analyses were conducted to determine the cellular identities of the hESC‐derived donor cells. HESC‐derived cells showed positive staining for the neuronal marker β3‐tubulin (TUBB3) (Figure 3B) as well as the glial marker S100β (Figure 4B). Transplanted cells within the DRG also revealed expression of the pan‐neuronal peripheral marker, human peripherin (Figures 3C and 4C), as well as DRG subtype markers, TRKA (2.5% ± 0.3% positive cells), TRKB (97.3% ± 2.1% positive cells) and TRKC (20.6% ± 1.9% positive cells) (Figure 5). Note that some donor cells may co‐express TRK receptors during early stages of differentiation, as described in DRG development.^40^ The donor neurons showed bipolar elongated processes, as expected for embryonic DRG sensory neurons (Figure 3C).^41^

One technical challenge of transplanting cells into the DRG is that the DRG is surrounded by connective tissue, which makes the penetration of the DRG using a glass capillary difficult. Instances of ectopically injected cells outside the DRG are documented in Figure 4. In these scenarios, we observed donor cells survival and differentiation to neurons and glia in the host tissue as well as migration along the DRG structure surface (Figure 4).

These data demonstrate that hESC‐derived cells are able to survive within the adult rat DRG region and show evidence of neuronal differentiation 2 weeks post‐transplantation. These results warrant further investigation to assess the longer‐term outcomes of transplanted cells within the DRG.

### 3.3 Survival, localization, and characterization of FRDA iPSC‐derived donor cells 8 weeks post‐transplantation

**FIGURE 6:**
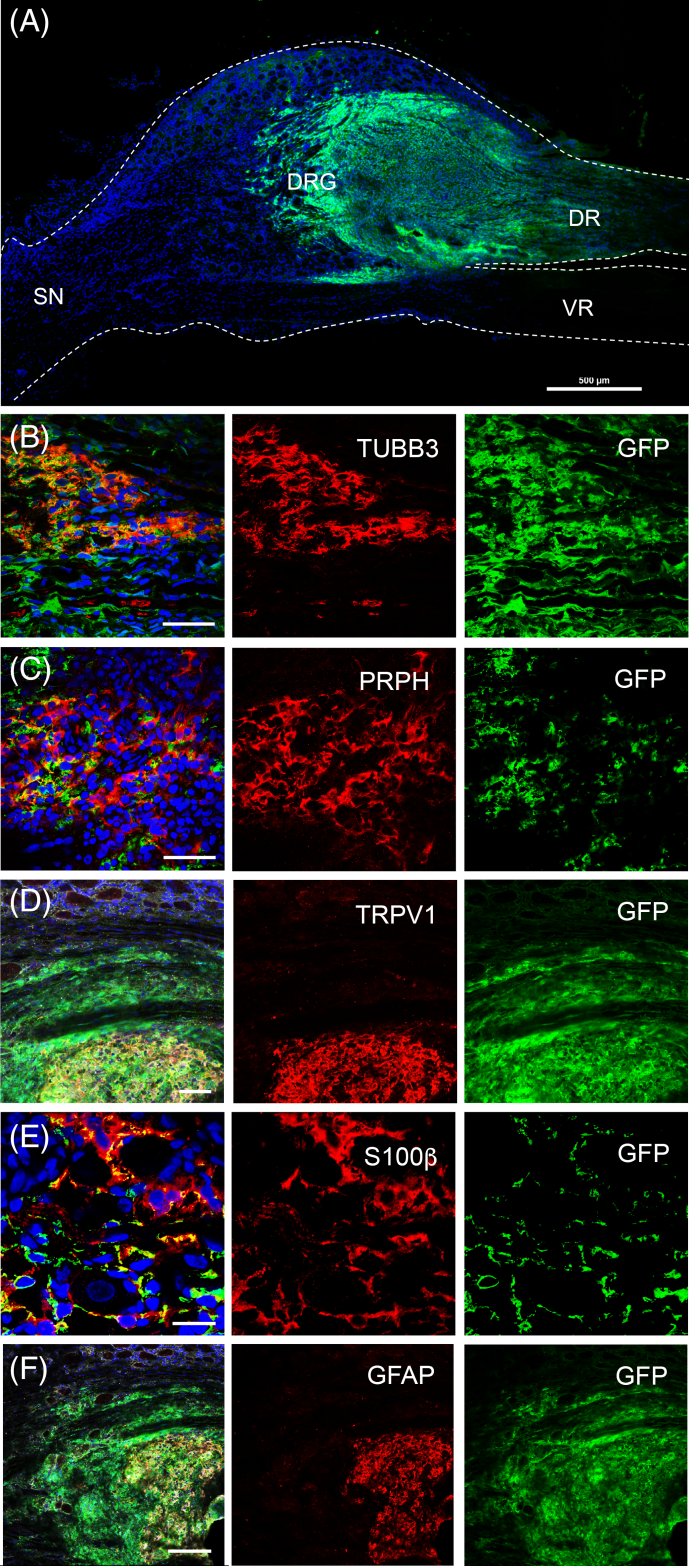
In vivo characterization of FA10‐GFP donor cells 8 weeks post‐transplantation into DRG of adult athymic rats. (A) FA10‐GFP^+^ cells after 8 weeks of transplantation. Immunostaining analyses revealed presence of FA10‐GFP^+^ cells positive for β3‐tubulin (B, red), peripherin (C, red) TRPV1 (D, red), S100β (E, red), and GFAP (F, red). Nuclei are shown in blue (DAPI). Scale bars: (A) 500 μm, (B, C, E) 50 μm, (D) 20 μm, (F) 100 μm. DR, dorsal root; GFAP, glial fibrillary acidic protein; SN, sciatic nerve; TRPV1, transient receptor potential cation channel, subfamily V, member 1; TUBB3, PRPH, human peripherin; S100β, S100 calcium binding protein B; VR, ventral root; β3‐tubulin

Given the positive results we obtained using hESC‐derived sensory neurons, we proceeded to examine the ability of FRDA iPSC‐derived sensory neurons to survive, differentiate and integrate in the DRG. For these studies we transplanted sensory neurospheres (NSP) derived from a GFP reporter FRDA iPSC line (FA10‐GFP) in the DRG at L4 level of the spinal cord of adult athymic “nude” rats. Transplanted animals were maintained for 8 weeks to examine longer‐term survival and integration of FRDA iPSC neurons in the DRG regions. Analyses of transplanted tissues at 8 weeks post‐transplantation showed a large number of GFP^+^ cells within the injected DRG region (46 000 cells, 1.5‐fold higher than the number of cells injected) (Figure 6A).

**FIGURE 7:**
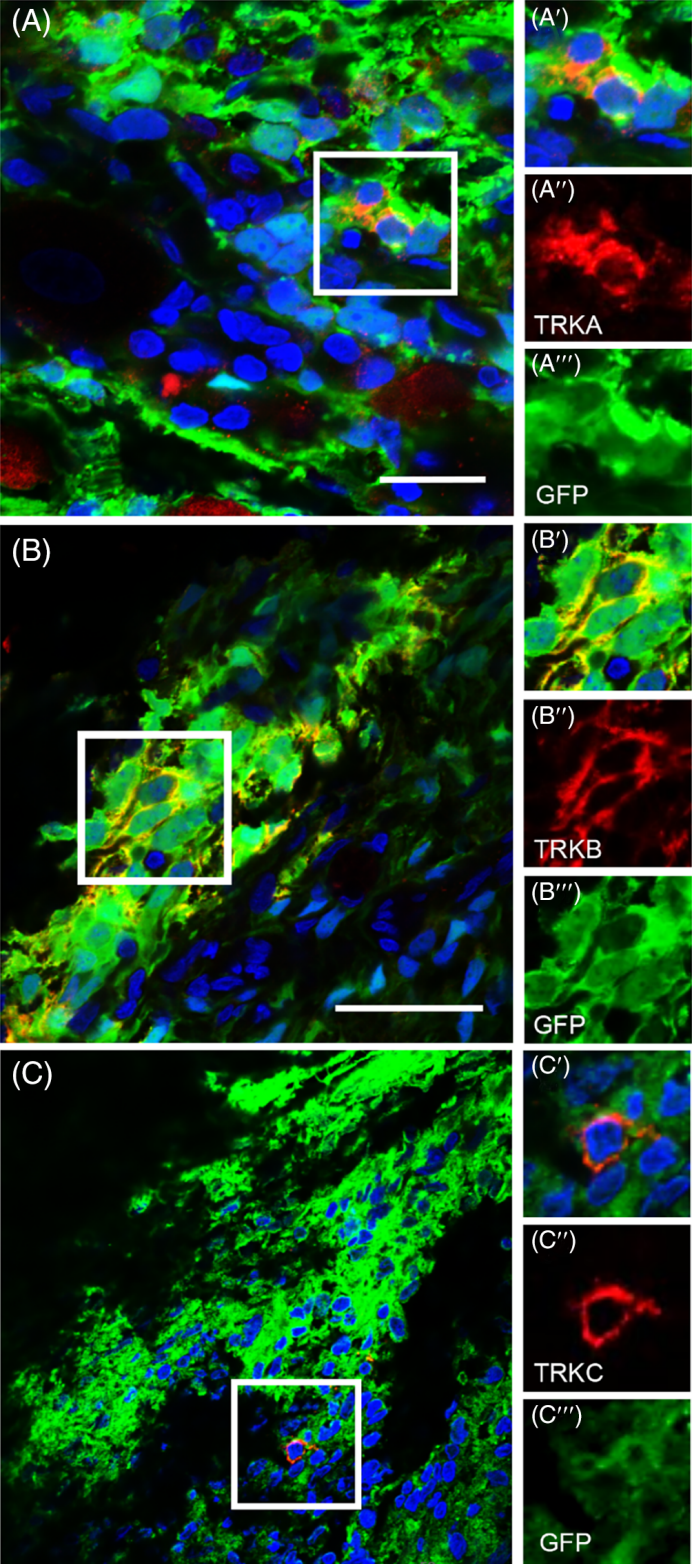
Expression of TRK receptors in FA10‐GFP^+^ iPSC‐derived donor cells 8 weeks post‐transplantation into DRG of adult athymic rats. Immunostaining analyses of transplanted DRGs identifies expression of hTRKA (A, A′, A″, red), hTRKB (B, B′, B″, red) and hTRKC (C, C′, C″, red), which are markers of nociceptive, mechanoreceptive and proprioceptive neurons, respectively. Nuclei are shown in blue (DAPI). Scale bars: (A, B) 20 μm, (C) 50 μm

We next proceeded to characterize phenotypes of the grafted cells histologically. FA10‐GFP^+^ cells showed expression of the neuronal marker, β3‐tubulin (TUBB3, Figure 6B). A large number of GFP^+^ cells also expressed the peripheral neuronal marker, peripherin (PRPH, Figure 6C) as well as sensory neuronal markers TRPV1 (Figure 6D), hTRKA (10.3% ± 5.4% positive cells) (Figure 7A), hTRKB (38.6% ± 5.2% positive cells) (Figure 7B) and hTRKC (20.2% ± 12.7% positive cells) (Figure 7C). In addition to neuronal markers, donor FA10‐GFP^+^ cells expressing glial markers, S100β and GFAP, were also detected (Figure 6E,F). Interestingly, human cells positive for the S100β marker surrounded the host sensory neurons (Figure 6E) similarly to that which occurs with endogenous S100β^+^ satellite glial cells.^42^ Overall, these results suggest FA iPSC‐derived cells showed capacity to differentiate in vivo into heterogeneous populations of neurons, particularly sensory neurons, as well as glia.

Another important aspect examined within the transplanted tissue was the presence of neuronal projections from the grafted cells, which extended into the spinal cord and along the endogenous neuronal tracts. Immunostaining analyses revealed FA10‐GFP^+^ expression throughout the DRG structure and in the dorsal root region, which connects the DRG with the spinal cord (see Supplementary Figure S5A). In contrast, no GFP^+^ cells were detected in the ventral root or in the region that forms part of the sciatic nerve (see Supplementary Figure S5B,C). These results were further confirmed by immunostaining analyses performed on sciatic nerve and spinal cord sections taken from the neighboring transplanted tissues (see Supplementary Figure S5C,D).

## 4 DISCUSSION

To the best of our knowledge, this is the first study showing transplantation of hPSC‐derived sensory neural progenitors into the DRG. Although transplantation of hPSC neural derivatives in the CNS have been widely investigated, only a few studies have been conducted to examine cell grafting in and around the DRG structure. Such examinations are pertinent for the treatment of peripheral neuropathies and/or neurodegenerative diseases, such as FRDA, whereby DRG sensory neuronal degeneration occurs. To this end, we generated a novel GFP‐labeled FRDA iPSC line. Both hESC and FRDA iPSC lines were differentiated to sensory neural progenitors in vitro. Immunostainings and RT‐qPCR analyses verified the presence of neurons expressing proprioceptive, mechanoreceptive and nociceptive neuronal markers. Transplantation of hPSC‐derived sensory neural progenitors into adult rat DRG regions revealed persistent expression of neuronal markers, including sensory markers, peripherin and TRK receptors, and glial differentiation markers. We also found that number of donor cells within the graft was increased relative to number of cells transplanted, suggesting the donor progenitor cells were capable of proliferating in vivo as well as differentiating to neuronal or glial lineages. Interestingly, at both 2 and 8 weeks post‐transplantation, the grafts showed relatively high proportion of TRKB expression whereas relatively fewer cells expressed TRKA. Although we have previously reported that the hPSC sensory differentiation protocol gives rise to similar proportions of TRKA, B and C expression in vitro,^37^ it is also well documented that HPSC sensory neuronal differentiation may be highly variable between experiments.^43^ The in vitro versus in vivo expression of hPSC‐derived sensory neuronal and glial markers may be addressed in future studies using higher numbers of transplanted animals and multiple hPSC lines. Overall, these findings provide evidence of the in vivo differentiation capacity of hPSC‐derived sensory neural progenitors within the adult DRG microenvironment and may be a promising therapeutic avenue for the treatment of PNS degenerative diseases.

Transplantation into DRG regions is technically challenging due to its small structure and anatomical location, residing under the spinal vertebrae. It is not surprising therefore that in some animals donor cells resided outside DRG regions, likely due to ectopic injection rather than extravasation from the DRG. Nevertheless, donor cells survived outside the DRG and maintained expression of neuronal and glial markers. Functional characterization of the transplanted donor neurons in vivo was not possible due to the difficulties obtaining intact DRG or DRG slices to perform electrophysiological analyses. Transplantation of Ca^2+^ reporter hPSC cell lines might provide the means to evaluate the activity of the transplanted cells. A similar method was discussed by Chen and Huang, whereby they investigated Ca^2+^ signals in neurons and neuron‐glia interactions in intact DRG with the help of fluorescent Ca^2+^ indicators.^44^ Our previously published studies showed hPSC‐derived sensory neurons are functional in vitro and respond to sensory stimuli, including heat, osmotic changes, and nociceptor agonists.^37^ Functional data of donor cells in vivo could also be provided in future studies by performing transplantation of hPSC‐derived sensory neurons in an animal model of peripheral neuropathy and assessing their sensory functions.

One of the primary and most severe sites of neurodegeneration that is consistently observed in FRDA is the DRG.^45^, ^46^, ^47^ However, neurodegeneration also occurs in other neuronal populations within the CNS and PNS and for some patients, degeneration is also observed in non‐neuronal tissues such as cardiac tissue.^48^ For this reason, it is considered that a combination of multiple therapeutic interventions may be needed to ameliorate FRDA. Clinical trials for cell replacement therapies are starting for certain neurodegeneration conditions, including Parkinson's Disease.^49^ Given that the DRG are clusters of regenerative neurons,^50^, ^51^, ^52^ exploring the possibility of cell replacement therapy in the DRG bears hope for treatment of FRDA. By showing long‐term survival of donor neurons within an adult host DRG region, our data work provides a significant step forward of such therapeutic approaches. Future studies should involve similar transplantation experiments with even longer follow up times to assess the capacity of these neurons for appropriate anatomical connectivity and should also be performed in FRDA rodent models using donor sensory neurons derived from corrected FRDA iPSC lines.

## 5 CONCLUSION

In summary, this report is one of the first to describe transplantation of hPSC‐derived sensory neurons into the adult rodent DRG. These studies are valuable for the understanding of hPSC‐derived neuron properties and functionalities in vivo, as well as for elucidating their therapeutic application as alternative therapy for treating peripheral neuropathies affecting sensory neurons of the DRG. Taken together, these findings contribute to our understanding of the feasibility of novel stem cell‐based therapies for treating peripheral neuropathies, such as FRDA.

## CONFLICT OF INTEREST

The authors declared no potential conflicts of interest.

## AUTHOR CONTRIBUTIONS

S.V.: conception and design, collection and/or assembly of data, data analysis and interpretation, manuscript writing, final approval of manuscript; S.F.: conception and design, collection and/or assembly of data, data analysis and interpretation, final approval of manuscript; S.E.H.: provision of study material, final approval of manuscript; S.Y.L., R.K.F., J.R.M.: collection and/or assembly of data, final approval of manuscript; K.D.A.: provision of study material, final approval of manuscript; W.N.: consultation of transplantation technique, final approval of manuscript; J.I.: data analysis and interpretation, final approval of manuscript; L.T.: conception and design, financial support, administrative support, provision of study material, data analysis and interpretation, final approval of manuscript; M.D.: conception and design, financial support, administrative support, provision of study material, collection and/or assembly of data, data analysis and interpretation, manuscript writing, final approval of manuscript.

## Supporting information


**Appendix S1**: Supporting information

## Data Availability

The data that support the findings of this study are available from the corresponding author upon reasonable request.
