# Supplementary material for: In vivo survival and differentiation of Friedreich ataxia iPSC‐derived sensory neurons transplanted in the adult dorsal root ganglia
Source: Stem Cells Transl Med. 2021 Mar 18;10(8):1157–69. doi: 10.1002/sctm.20-0334 (PMC8284774; doi:10.1002/sctm.20-0334)
Supplement: Supplementary file 1 — Appendix S1: Supporting information [file SCT3-10-1157-s001.docx]

**Supporting Information**

**In vivo survival and differentiation of Friedreich ataxia iPSC-derived sensory neurons transplanted in the adult dorsal root ganglia**

Serena Viventi ^1, 2^, Stefano Frausin ^3^, Sara E. Howden ^4^, Shiang Y. Lim ^5^, Rocio K. Finol-Urdaneta ^6^, Jeffrey R. McArthur ^6^, Kwaku Dad Abu-Bonsrah ^1,4^, Wayne Ng ^7^, Jason Ivanusic ^8^, Lachlan Thompson ^3^ and Mirella Dottori ^1, 2, 6, 8 *^

^1^ Centre for Neural Engineering, The University of Melbourne, Australia

^2^ Department of Biomedical Engineering, The University of Melbourne, Australia.

^3^ The Florey Institute of Neuroscience and Mental Health, Australia**.**

^4^ The Murdoch Childrens Research Institute, Royal Childrens Hospital, Australia

^5^ O'Brien Institute Department, St Vincent's Institute of Medical Research, Department of Surgery, University of Melbourne, St Vincent Hospital, Australia

^6^ Illawarra Health and Medical Research Institute, University of Wollongong, Australia

^7^ Austin Hospital, Australia

^8^ Department of Anatomy and Neuroscience, The University of Melbourne, Australia

* Corresponding author:

Mirella Dottori, PhD

Illawarra Health and Medical Research Institute

Building 32, University of Wollongong, NSW 2522 Australia

Ph: +61 2 4221 5233

Email: mdottori@uow.edu.au

**
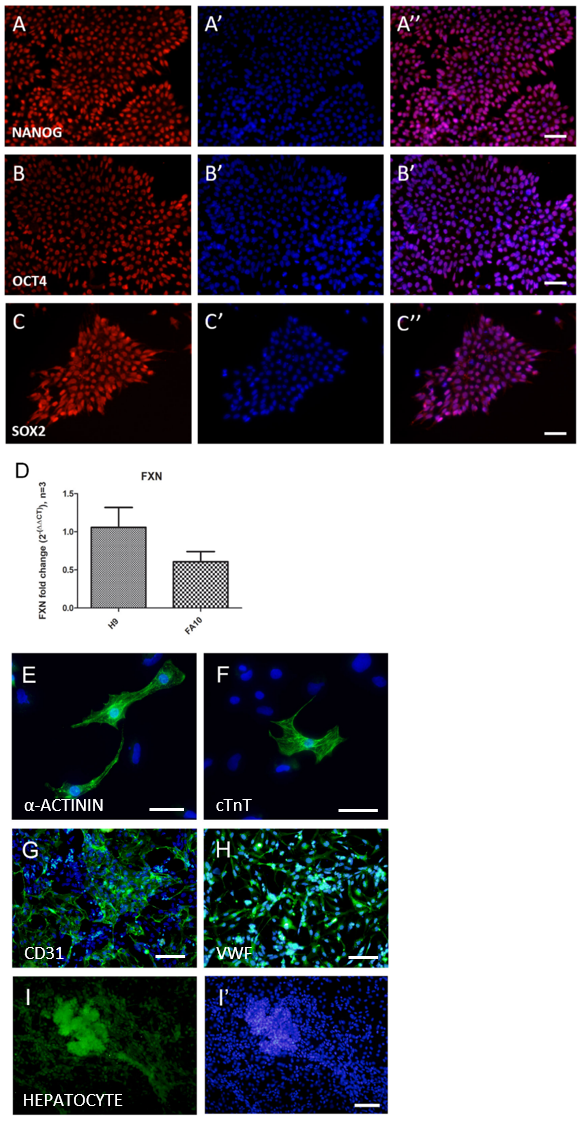
**

**Figure S1. Characterization of FRDA iPSC line. (A, B, C)** Expression of pluripotent stem cell markers NANOG (A, A’’, red), OCT4 (B, B’’, red), SOX2 (C, C’’, red). **(D)** Q-PCR analyses of *FXN* expression in undifferentiated hESC and FRDA iPSC lines. Data presented as mean with error bars representing standard error of mean (SEM). n=3 independent experiments, each experiment has n=3 replicate samples. T-test was used to assess significance. **(E, F)** FRDA iPSC differentiation to mesoderm. FRDA iPSCs-derived cardiomyocytes are immunoreactive for cardiac α-ACTININ (E) and cTnT (F). FRDA iPSC-derived endothelial cells are immunoreactive for CD31 and VWF. **(I)** FRDA iPSC differentiation to endoderm. FRDA iPSC endoderm progenitors show positivity for human hepatocyte-specific antigen (OCH1E5)**.** Dapi nuclei are shown in blue. Scale bars: (A, B, C, E, F) 50 μm, (G, H, I) 100 μm.


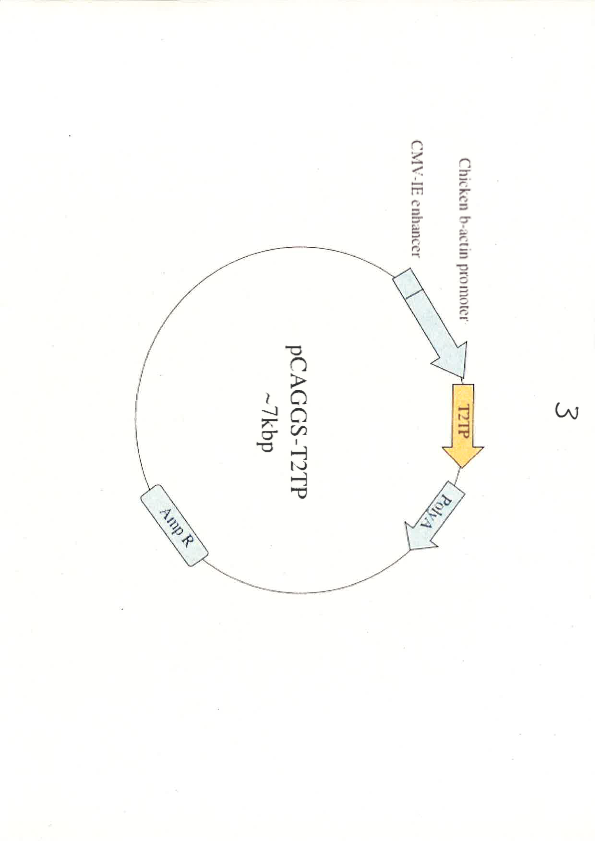

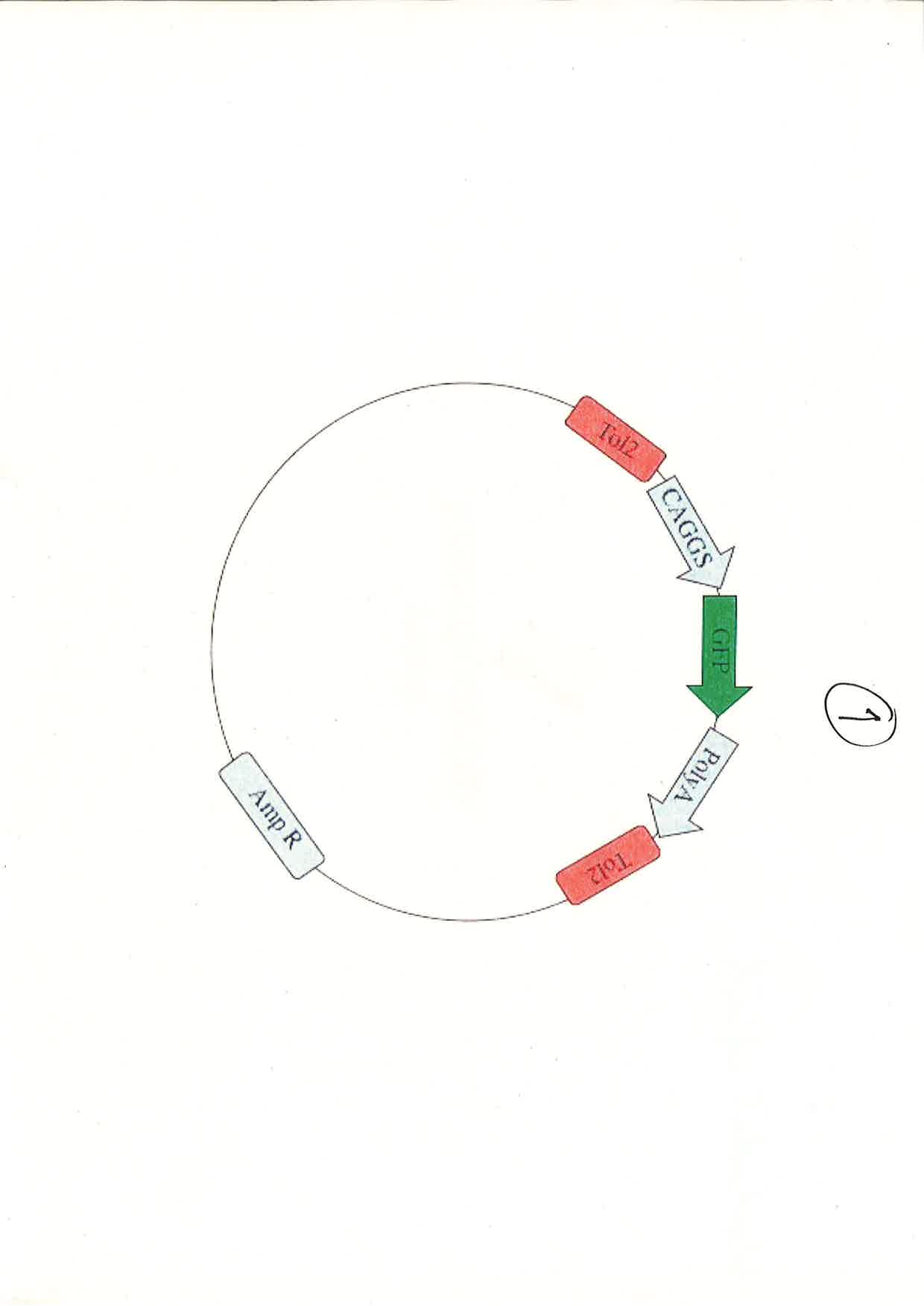


**Figure S2.** T2K-GFP plasmid (A) and T2TP enzyme (B) used for transfection of FRDA iPSC cell line, FA10.

**
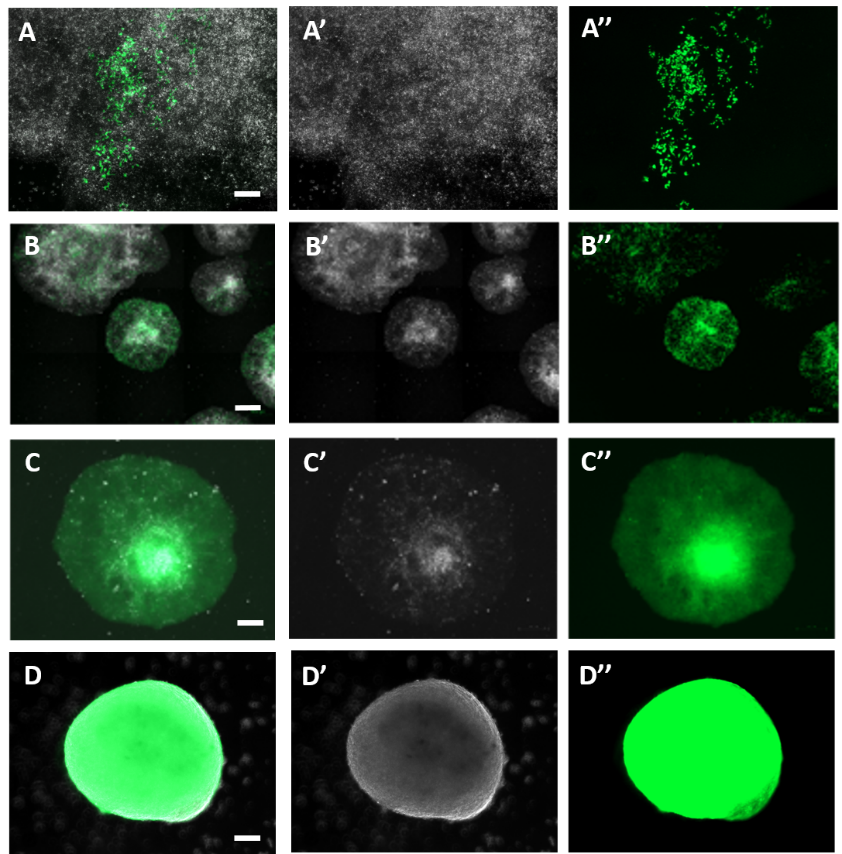
**

**Figure S3. Generation of FA10-GFP cell line.** Fluorescent (A-D, A’’-D’’), brightfield (A-D, A’-D’) and merged (A-D) images of FA10-GFP iPSC and NSP. (A, A’, A’’) Images show presence of GFP-expressing positive FA10 cells 3 days following electroporation of the GFP-expression plasmid. (B, B’, B’’) Formation of partially GFP-expressing FA10 colonies after manual selection. (C, C’, C’’) Formation of full-GFP-expressing FA10 colony after mechanical selection and expansion. (D, D’, D’’) GFP-expressing FA10-derived sensory 3 week old NSP. Scale bars: (A, C and D) 200 μm, (B) 500 μm


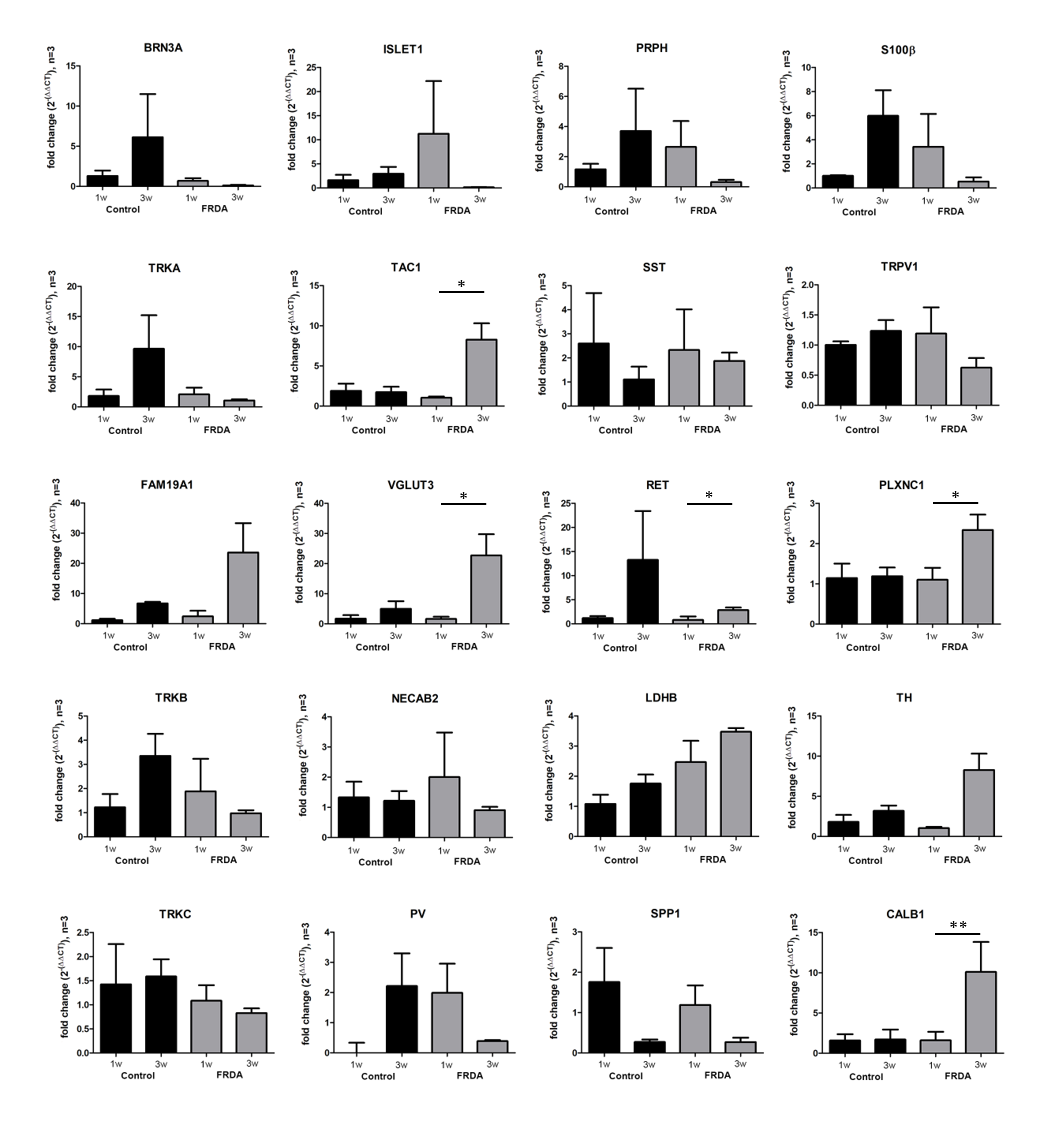


**Figure S4. Q-PCR data showing expression of sensory neuronal markers in hESC and FRDA iPSC lines at 3 weeks of differentiation relative to 1 week.** Data presented as mean with error bars representing standard error of mean (SEM). n=3 independent experiments, each experiment has n=3 replicate samples. T-test was used to assess significance. * p<0.05 and **p<0.01.


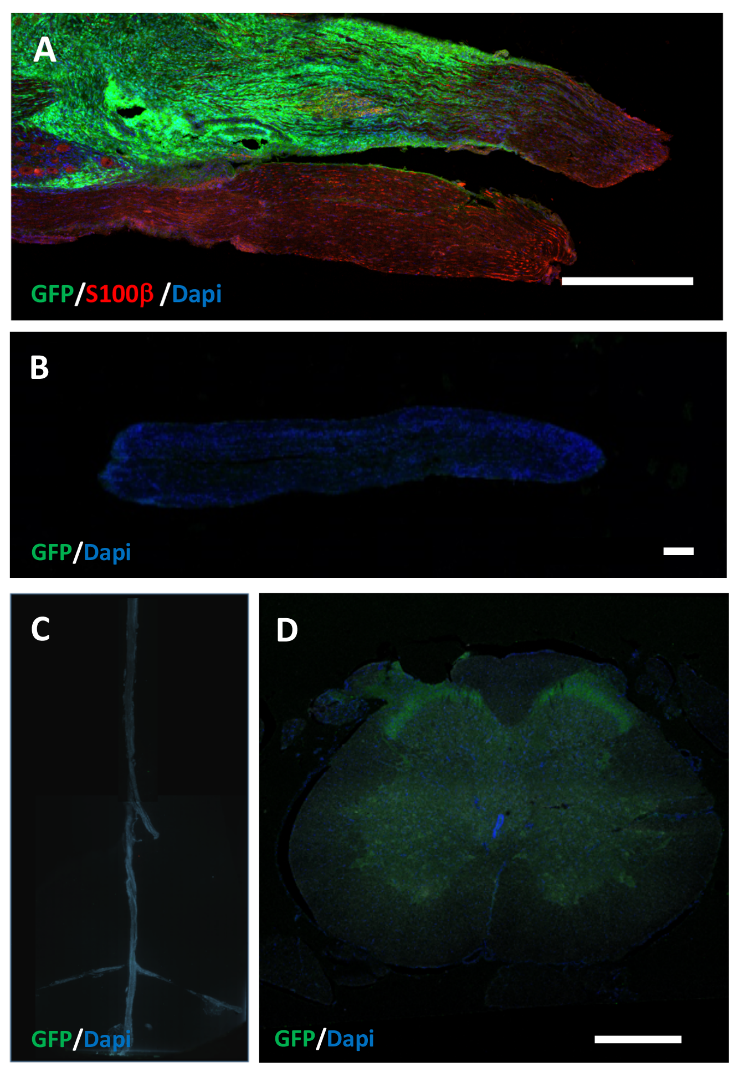


**Figure S5. Immunohistochemical analyses of sciatic and spinal cord tissues in athymic rats transplanted with FA10-GFP^+^ derived cells at 8 weeks post-transplantation.** FA10-GFP^+^ donor cells were identified in the dorsal root of the transplanted DRG (A, green). S-100β (A, red). GFP^+^ cells were not detected either in the dorsal root region proximal to the spinal cord (B), nor in the sciatic nerve which is connected to the DRG (C). No GFP^+^ cells were found within the spinal cord (D). Dapi nuclei are shown in blue. Scale bars: (A, D) 500 μm, (B) 1000 μm. Note that the image shown in ‘C’ is composed of two images joint as one in order to show the entire tissue section.
